# Context matters: physical activity in the Western Pacific region

**DOI:** 10.1016/j.lanwpc.2024.101190

**Published:** 2024-08-30

**Authors:** The Lancet Regional Health – Western Pacific

The Olympics showcase amazing athleticism and sportsmanship. The Paris 2024 Olympics includes 32 different sports such as athletics and swimming, as well as more niche sports like skateboarding and breakdancing. During these Olympics, Māori and Pasifika communities have called for indigenous ways of moving, a vital component of their wellbeing, to be considered as sport and physical activity. This has brought to the forefront the important role of culture and context in influencing practices surrounding physical activity to optimise health outcomes.

Globally, 23% of adults and 81% school-going adolescents do not participate in sufficient physical activity. Global trends show increasing prevalence of insufficient physical activity among adults: 28.6% in 2010 to 31.3% in 2022. Insufficient physical activity is particularly prominent in high-income Asia–Pacific regions (48%) and south Asia (45.4%) compared to Oceania (13.6%), which is on track to meet the global target of a 15% reduction in insufficient physical activity by 2030. Contextual factors, including physical activity type (eg, incidental and occupational), as well as rapid economic development and urbanisation in high-income Asia–Pacific regions may account for this variability. Regional variation may also reflect more striking benefits of health policy advances among people with initially lower levels of physical activity, highlighting the importance of research and interventions targeting underserved populations. For example, the Western Pacific Regional Action Plan for the Prevention and Control of Noncommunicable Diseases identifies physical inactivity as a key risk factor for a range of non-communicable diseases (NCDs), such as stroke, diabetes and cancer. Recommended actions for Member States include implementation of public health campaigns to promote the benefits of physical activity and healthy diets, particularly from a life-course approach.

Early childhood development is characterised by rapid physical, psychological, and social growth. As such, early childhood is an important window of opportunity to develop healthy habits to prevent and reduce risk factors common to many NCDs. The Asia–Pacific consensus statement on integrated 24-hour activity for the early years provides guidelines for physical activity, sedentary behaviour, and sleep for children under the age of 5 years. For example, pre-schoolers (age 3–4 years) are recommended to accumulate at least 180 min of physical activity, limit sedentary time to less than 1 h per day, get 10–13 h of daily sleep, and eat more nutrient-dense food. Importantly, these guidelines refer to a range of physical activities depending on age and motor skills (eg, locomotor, object control among toddlers) and built environment approaches to promote physical activity (eg, childcare infrastructure that includes more open space to promote physical activity). Adherence to these guidelines could lead to several benefits such as improved fitness, mental wellbeing, and quality of life. However, there is still much work to be done to integrate physical and mental wellbeing in public health policies and strategies. This is a major barrier to a more holistic understanding of health issues and their prevention and management.

It is well established that physical activity is important for physical and mental wellbeing. The comorbidity between NCDs and mental health disorders is associated with worse health outcomes, more complex care needs and clinical management, and higher health-care costs. However, this relationship is often overlooked. This is concerning given that mental health disorders are a major contributor to the burden of NCDs in the Western Pacific region. More than 215 million people have a mental health condition in the region, which is amplified by population ageing, rapid economic development, urbanisation, and climate change. There is increasing evidence for the treatment of depression via lifestyle therapies that focus on diet and physical activity. The CALM randomised trial showed that lifestyle therapy targeting nutrition and physical activity via a dietitian and exercise physiologist was non-inferior to the mainstay of depression treatment, psychotherapy (cognitive behavioural therapy). The SMARTDiabetes cluster randomised controlled trial conducted in China suggested that a multifaceted digital health intervention made up of a self-management app for patients and family health promotors and clinical decision support for primary care doctors was associated with improved glycaemic control. Local contextualisation of the intervention involving implementation and integration into the existing primary health-care system, and active engagement of patients, family members, and health-care providers were key factors contributing to its effectiveness, particularly in rural areas.

Contextualisation is imperative to effectively transfer evidence into locally relevant and impactful public health policies and programmes. Research on physical activity should be more solution focused, employ cross-sectoral approaches, and consider populations that should be prioritised, as well as considering co-benefits such as long-term positive mental and social outcomes. For older people, the benefits of physical activity include reduced risk of NCDs, prevention of ageing-related physical decline, as well as improved mental wellbeing. The rapidly ageing population in the Western Pacific region requires population-level programmes and strategies tailored to the local needs, resources, and finances of older people. This includes health promotion strategies that minimise the adverse impact of ageism and focus on the additional benefits of physical activity for older adults, specifically improved mental health, social connectedness, and independence, which are important for quality of life. Li and colleagues found that higher levels of occupational moderate-to-vigorous physical activity were associated with up to 40% lower risk of all-cause and cardiovascular disease-specific mortality among people living in rural areas compared to those in urban areas. This difference may reflect socioeconomic differences, whereby the vast majority of adults participating in occupational moderate-to-vigorous physical activity were in rural rather than urban settings. In urban areas, limited access to quality health care among people with lower socioeconomic status might attenuate the protective benefits of physical activity. This again demonstrates the importance of considering different types of physical activity as well as socioeconomic factors associated with access to and participation in physical activity. Population-specific public health policies and strategies targeting physical activity are needed to ensure access to high quality preventive health care.

To fully realise the benefits of healthy lifestyle interventions, we need more inclusive and holistic health oriented public health policies and strategies. Prioritising physical activity from an early age is critical for long-term wellbeing. Physical and mental wellbeing go hand-in-hand. Integrating physical and mental wellbeing NCD policies and strategies that are tailored to the unique needs and context of communities is essential to achieve the highest level of health for all people.

